# MISCAST: MIssense variant to protein StruCture Analysis web SuiTe

**DOI:** 10.1093/nar/gkaa361

**Published:** 2020-05-13

**Authors:** Sumaiya Iqbal, David Hoksza, Eduardo Pérez-Palma, Patrick May, Jakob B Jespersen, Shehab S Ahmed, Zaara T Rifat, Henrike O Heyne, M Sohel Rahman, Jeffrey R Cottrell, Florence F Wagner, Mark J Daly, Arthur J Campbell, Dennis Lal

**Affiliations:** Center for Development of Therapeutics, Broad Institute of MIT and Harvard, Cambridge, MA 02142, USA; Stanley Center for Psychiatric Research, Broad Institute of MIT and Harvard, Cambridge, MA 02142, USA; Analytic and Translational Genetics Unit, Massachusetts General Hospital, Boston, MA 02114, USA; Luxembourg Centre for Systems Biomedicine, University of Luxembourg, Esch-sur-Alzette, Luxembourg; Department of Software Engineering, Faculty of Mathematics and Physics, Charles University, Prague, Czech Republic; Genomic Medicine Institute, Lerner Research Institute Cleveland Clinic, Cleveland, OH 44195, USA; Luxembourg Centre for Systems Biomedicine, University of Luxembourg, Esch-sur-Alzette, Luxembourg; Department of Bio and Health Informatics, Technical University of Denmark, Lyngby, Denmark; Computer Science and Engineering, Bangladesh University of Engineering and Technology, ECE Building, West Palashi, Dhaka-1205, Bangladesh; Computer Science and Engineering, Bangladesh University of Engineering and Technology, ECE Building, West Palashi, Dhaka-1205, Bangladesh; Stanley Center for Psychiatric Research, Broad Institute of MIT and Harvard, Cambridge, MA 02142, USA; Analytic and Translational Genetics Unit, Massachusetts General Hospital, Boston, MA 02114, USA; Institute for Molecular Medicine Finland (FIMM), University of Helsinki, 00100 Helsinki, Finland; Computer Science and Engineering, Bangladesh University of Engineering and Technology, ECE Building, West Palashi, Dhaka-1205, Bangladesh; Stanley Center for Psychiatric Research, Broad Institute of MIT and Harvard, Cambridge, MA 02142, USA; Center for Development of Therapeutics, Broad Institute of MIT and Harvard, Cambridge, MA 02142, USA; Stanley Center for Psychiatric Research, Broad Institute of MIT and Harvard, Cambridge, MA 02142, USA; Stanley Center for Psychiatric Research, Broad Institute of MIT and Harvard, Cambridge, MA 02142, USA; Analytic and Translational Genetics Unit, Massachusetts General Hospital, Boston, MA 02114, USA; Institute for Molecular Medicine Finland (FIMM), University of Helsinki, 00100 Helsinki, Finland; Center for Development of Therapeutics, Broad Institute of MIT and Harvard, Cambridge, MA 02142, USA; Stanley Center for Psychiatric Research, Broad Institute of MIT and Harvard, Cambridge, MA 02142, USA; Stanley Center for Psychiatric Research, Broad Institute of MIT and Harvard, Cambridge, MA 02142, USA; Genomic Medicine Institute, Lerner Research Institute Cleveland Clinic, Cleveland, OH 44195, USA; Cologne Center for Genomics, University of Cologne, Cologne, Germany; Epilepsy Center, Neurological Institute, Cleveland Clinic, Cleveland, OH 44195, USA

## Abstract

Human genome sequencing efforts have greatly expanded, and a plethora of missense variants identified both in patients and in the general population is now publicly accessible. Interpretation of the molecular-level effect of missense variants, however, remains challenging and requires a particular investigation of amino acid substitutions in the context of protein structure and function. Answers to questions like ‘Is a variant perturbing a site involved in key macromolecular interactions and/or cellular signaling?’, or ‘Is a variant changing an amino acid located at the protein core or part of a cluster of known pathogenic mutations in 3D?’ are crucial. Motivated by these needs, we developed MISCAST (missense variant to protein structure analysis web suite; http://miscast.broadinstitute.org/). MISCAST is an interactive and user-friendly web server to visualize and analyze missense variants in protein sequence and structure space. Additionally, a comprehensive set of protein structural and functional features have been aggregated in MISCAST from multiple databases, and displayed on structures alongside the variants to provide users with the biological context of the variant location in an integrated platform. We further made the annotated data and protein structures readily downloadable from MISCAST to foster advanced offline analysis of missense variants by a wide biological community.

## INTRODUCTION

Large-scale genome and exome sequencing projects have identified millions of single amino acid-altering missense variants in the relatively healthy general population (1). In addition, the growing use of genetic screening in clinical practice has revealed a large number of missense variants as the molecular basis of many diseases (2,3). With the aim of persistent data sharing and subsequent analysis, the variant level data are now systematically aggregated in many public databases such as ExAC (Exome Aggregation Consortium) (4), gnomAD (Genome Aggregation Database) (5), ClinVar (6), HGMD (Human Gene Mutation Database) (7) and OMIM (8). Concomitantly with the growth of genomics data, rapid advances in electron microscopy and other experimental techniques for determining macromolecular structure have produced thousands of protein structures that are accessible through resources like RCSB PDB (9) and PDBe (10). These data now offer an opportunity to study the molecular-level effect of missense variants in the context of protein structure and function.

Given that the damaging and/or neutral consequences of missense variants are reportedly associated with the structural properties of the affected amino acids (11,12), studying these variants’ effect on protein structure represents a growing research area. However, linking single nucleotide variations (SNVs) from VCF files to the location of the amino acid substitution on the structure (stored in PDB files, https://www.rcsb.org/) is still challenging. The formats of VCF and PDB files are largely different and their integration requires a bioinformatic skillset, ad hoc computing, and expertise in processing both genomics and structural data. Nonetheless, resources and tools have been developed to map missense variants on protein structures such as ASTRID (http://astrid.icompbio.net/), mutation3D (13), COSMIC-3D (14), VarSite (15), VarMap (16), PhyreRisk (17). While some of these tools provide the user with still graphics of protein structures with pre-mapped variants (e.g. ASTRID), some allow users to input variants and view them on the structure (mutation3D, COSMIC-3D, PhyreRisk, VarMap, etc.). Notably, some studies went beyond the mapping of variants onto the experimentally-solved structures only, and incorporated structure models and alternative transcripts to cover nearly the full proteome (e.g. PhyreRisk, VarMap). However, an integrated visual analytic platform to display variants and biologically-relevant features of residues on protein sequence and structure in a common web environment is not available. Recently developed 3DBIONOTES-WS server (18) performs concurrent sequence-structure visualization, which, however, does not allow to export the annotated data and 3D structures, limiting the opportunity for downstream analysis. To address these challenges, we have developed MISCAST: MIssense variant to protein StruCture Analysis web SuiTe (http://miscast.broadinstitute.org/).

MISCAST is an interactive web server where protein sequences are pre-annotated with disease-associated and population variants alongside a rich collection of biological context (structural, physicochemical and/or functional protein features), which are simultaneously overlaid on protein structures. The purpose of MISCAST is to support the scientific community in performing a quick, online inspection of missense variants on protein structures as well as allow users to download the structures annotated with variants and features to perform advanced offline investigation in PyMOL (https://pymol.org/2/).

## MATERIALS AND METHODS

### Collecting and filtering missense variants

**Figure 1.**
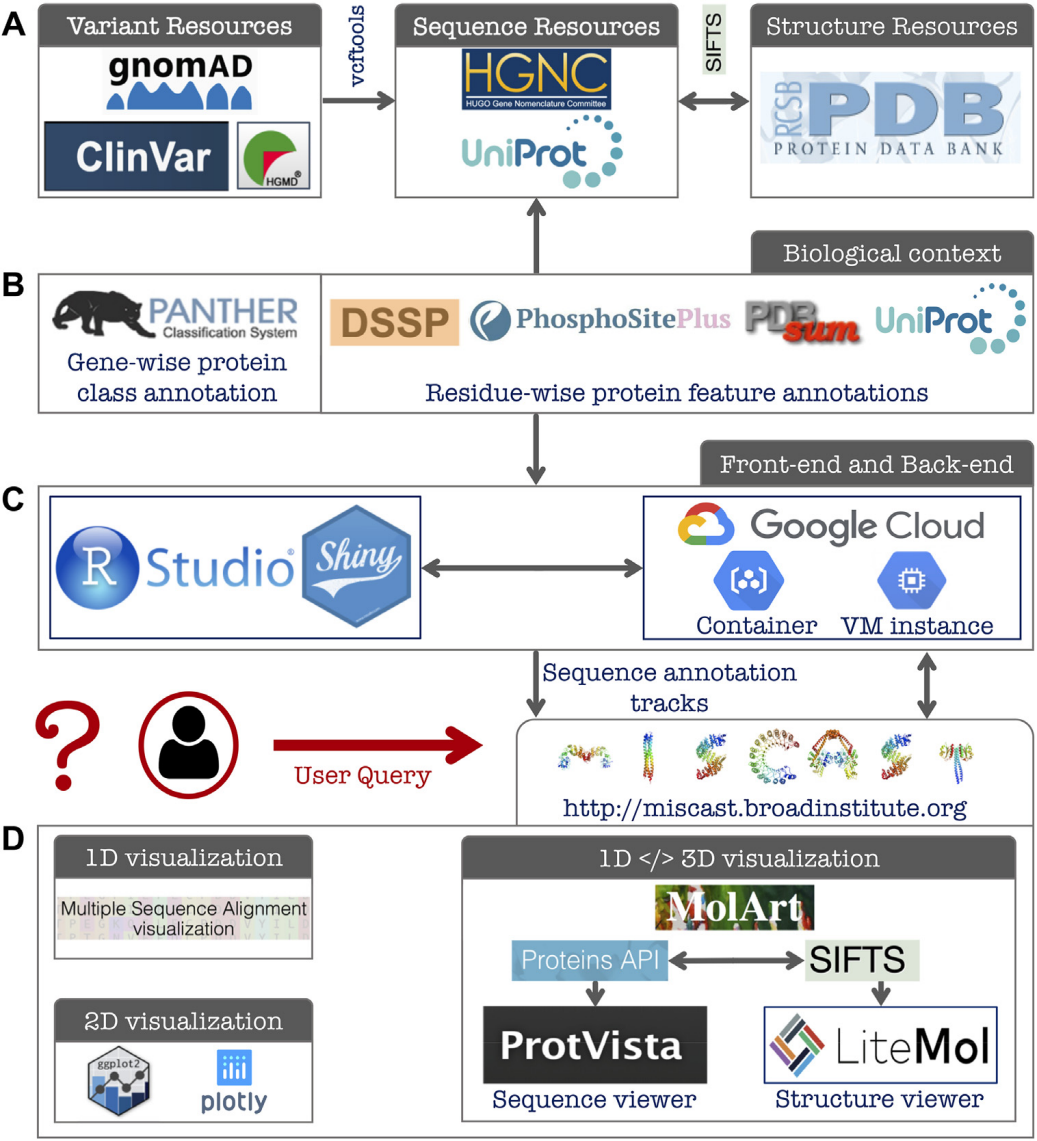
MISCAST architecture, main modules, and data flow. (**A**) Online resources used for collecting missense variants, gene symbols, protein sequences and structures. (**B**) Databases searched to aggregate gene- and residue-wise biological context annotations. (**C**) MISCAST development using Shiny R package and deployment using Google Cloud services. (**D**) Three main visualization schemes of MISCAST. Missense variants and protein features are displayed in 1D, 2D and concurrent 1D </> 3D views.

Missense variants in the general population were retrieved from the gnomAD database, public release 2.1.1 (Figure 1A). Consolidated files for all available exomes and genomes were downloaded as VCFs from http://gnomad.broadinstitute.org/downloads. Missense variants for canonical transcripts were extracted using the vcftools (19) and filtered based on the gnomAD standard quality control flag (‘PASS’). The disease-associated missense variants were collected from two databases: ClinVar (October, 2019 release) and HGMD® (professional release 2018.4). ClinVar data were filtered to keep variants with ‘Pathogenic’ and/or ‘Likely-Pathogenic’ clinical consequences. At the same time, the HGMD data were filtered to keep the high confidence disease-associated variants (confidence = ‘HIGH’, variantType = ‘DM’ or disease mutation). All annotations refer to the human reference genome version GRCh37.p13/hg19. Variants annotated to non-canonical transcripts were not considered.

In this paper and on the MISCAST web server, the variants obtained from gnomAD database, and that from ClinVar and HGMD databases are referred to as ‘population’ and ‘pathogenic’ variants, respectively.

### Collecting protein sequences, structures, functional-class annotations

All available experimentally-solved human 3D structures (in full or chimeric) were collected from the PDB, representing proteins encoded by 5850 genes. We filtered out 943 genes for which the canonical protein isoform sequences are not translated from the canonical transcripts. Out of the remaining 4907 genes, pathogenic missense variants were available for 1685 genes (ClinVar and/or HGMD databases). We further filtered out 355 genes for which no pathogenic variant was mappable onto the structures of the encoded proteins. Finally, we obtained 1330 genes, harboring in total 406,449 population and 54 137 pathogenic variants. For the vast majority of the genes (1322 out of 1330), the variants correspond to the Ensembl canonical transcripts while the RefSeq transcripts were used for the remaining genes. These variants are mapped onto 17 953 protein structures upon retrieving the residues’ positions on the structure from the SIFTS database (20) (Figure 1A). We then annotated the 1330 genes with encoded protein’s functional class information, collected from the PANTHER (19) database. The lists of genes, transcripts, and protein class annotations are available at https://github.com/dlal-group/MISCASTv1.0.

### Collecting protein features

We collected amino acid-wise annotations of forty protein features (a combination of structural, physicochemical and functional features) grouped into seven main categories: (i) 3-class secondary structures; (ii) 8-class secondary structures; (iii) residue exposure levels; (iv) physicochemical properties of amino acids; (v) protein-protein interactions; (vi) post-translational modifications (PTMs) and (vii) functional features. The secondary structure and solvent-exposure of amino acids were calculated using the DSSP program (22). Protein–protein interactions, PTMs, and functional features were obtained from the PDBsum (23), PhosphoSitePlus (24) and UniProt (25) databases, respectively (Figure 1B). The detailed feature mining and ascertainment procedure are described in the MISCAST (‘Documentation’) web server.

### Implementation of MISCAST

MISCAST is developed as a fully browser-based web application using the Shiny R package (https://shiny.rstudio.com/) in RStudio development environment (Figure 1C). Shiny can interactively translate queries from the user-end into HTML code, perform data analysis and computation in the back-end, and can display the results on the browser page in an extended fashion using CSS themes and JavaScript widgets. The annotation tracks (missense variants and protein features) per gene were assembled as tab-delimited text files (available at https://github.com/dlal-group/MISCASTv1.0). The summary reports on the effect of missense variants are dynamically generated by language processing, embedded in the R code written for MISCAST server. All the data and codes were uploaded as a self-standing container with Google Cloud services, which was then deployed into a Google Virtual Machine (VM). MISCAST is compatible with all commonly used internet browsers—with no installation or login requirement.

MISCAST leverages multiple JavaScript plugins to display missense variants and feature annotations on protein sequences and structures as informative visuals (Figure 1 D). The multiple sequence alignment (MSA) viewer (26) is used to visualize the protein sequence. All two-dimensional plots are generated using the R libraries, ggplot2 (27) and plotly (https://plot.ly). The concomitant visualization of annotations on sequence and structure was enabled by the MolArt tool (28). For a given gene, MolArt retrieves the available structure identifiers for the encoded protein using the Proteins API (29), obtains the sequence-structure mapping from SIFTS (20), and then displays the annotations on sequence and structure using ProtVista (30) and LiteMol (31) plugins, respectively. All the annotation tracks (pathogenic and population variants and forty protein features) for sequence (amino acid wise, as tab-delimited text file) and structure (as PyMOL file) are exportable. Further, the graphical and textual results are readily downloadable to use for scientific publication, presentation or downstream analysis.

## RESULTS AND CASE STUDY

### MISCAST web server

MISCAST is an interactive web server for integrated visualization of missense variants on the amino acid sequence and 3D structure of proteins. The current version of MISCAST includes all human genes with at least one pathogenic variant (ClinVar and HGMD databases) and those from the general population (gnomAD database) mappable onto an experimentally-solved 3D structure. Additionally, MISCAST includes residue-wise annotations of protein features reporting on amino acids’ chemical properties (e.g. aromatic versus charged or polar), structural context (α-helix, β- sheet, participation in hydrogen bonds, etc.) and their role in protein function (i.e. involvement in an enzyme’s active site, cellular signaling, etc.). These features are concomitantly viewable on protein sequences and structures alongside the missense variants, allowing users to examine the effect of variants considering the features of the affected residue.

MISCAST also shows annotations of the genes with protein-class information (kinases, transporters etc.) to provide the user with function-specific insights e.g. Is a variant of a gene encoding for a kinase perturbing the catalytic 3D site that is essential for the protein’s enzymatic activity? Finally, MISCAST highlights protein features that are associated with (or enriched for) pathogenic and population missense variants (32) *in general* (‘All protein classes’) and for specific groups of genes (i.e. encoding for a protein class).

**Figure 2.**
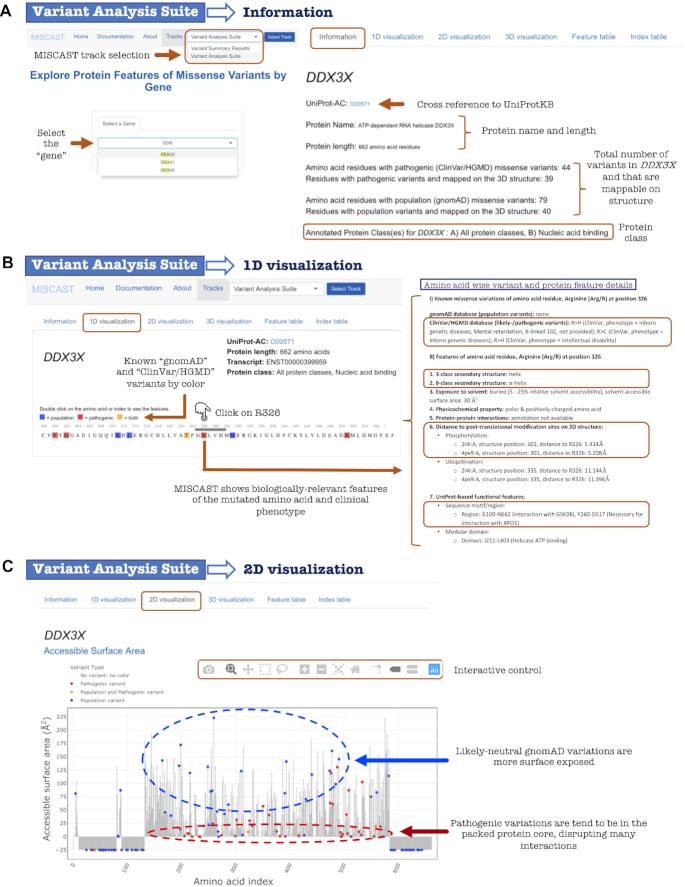
Selected visual and textual output of the Variant Analysis Suite track of MISCAST web server for the discussed case study. (**A**) Selection of a gene (*DDX3X*) opens up the ‘Information page’, displaying general overview of the encoded protein. (**B**) 1D visualization page to explore amino acid-wise missense variants alongside biologically-relevant protein feature annotations. (**C**) 2D visualization page to display missense variants in the context of feature annotations for the full protein sequence.

The homepage of MISCAST has two main navigation tracks: (i) variant analysis suite: enables inspection and analysis of variants upon querying by a gene symbol; (2) variant summary report: provides a report and an index describing the effect of missense variants based on the properties of the altered amino acids. At any point in the exploration, users can switch between the tracks from the ‘Select Track’ drop-down menu in the top navigation panel (Figure 2A).

### Variant analysis suite: information

Query by an HGNC gene symbol from the Variant Analysis Suite opens up the ‘Information’ page for that gene showing the UniProt accession (hyper-linked to UniProtKB), name and length of the encoded protein, variant counts, and the protein’s functional class information.

For example (Figure 2A), querying for *DDX3X* will show that it encodes for a 662-residue long ATP-dependent RNA helicase, which is a nucleic acid-binding protein. ClinVar and HGMD databases jointly record 44 missense variants of *DDX3X*; however, due to the partial coverage of the protein in structure, 39 of those (about 89%) were mappable on the structure. On the other hand, only about 50% (out of 79) of all missense variants of *DDX3X* obtained from gnomAD database were mappable on the protein structure. Here, users will also find the protein features associated with pathogenic and population missense variants *in general* (‘All protein classes’) and specifically for the class which *DDX3X* is annotated with, i.e. nucleic acid-binding protein. For example, the α-helical residues are particularly enriched with pathogenic variants of nucleic acid-binding proteins.

### Variant Analysis Suite: 1D visualization

The purpose of the 1D visualization page is to display the missense variants and functionally-relevant protein features at the amino acid level. Triggered by the query by gene, the page first shows the encoded protein sequence in the MSA viewer (Figure 2 B). Using the gray navigation bar, users can see the full sequence with residues colored according to the presence of pathogenic (red), population (blue) or both types (orange) of variants. This color scheme has been consistently maintained in every page of MISCAST to indicate the missense variant types. The sequence viewer is interactive: Users can double-click on the amino acid, which will in turn expand a panel below displaying the features of the selected amino acid and details of the available pathogenic (associated phenotype) and population (allele count and frequency values) variants.

Continuing with the example of *DDX3X* (Figure 2B), clicking on the Arginine (Arg/R) at position 326 (R326) will show that two different R326 mutations have been recorded as pathogenic in ClinVar: p.Arg326His (R > H) is associated with mental retardation and intellectual disability and p.Arg326Cys (R > C) is associated with inborn genetic diseases. Relevant protein features of R326 are also displayed, such as it forms an α-helix, it is close (∼5 Å) to a phosphorylation site in 3D despite being distant in sequence (25 residues gap), and is located in a region interacting with many other proteins (GSK3B, XPO1). These features alongside the variant information can guide the user to generate an informed hypothesis on the impact of a particular amino acid substitution.

### Variant analysis suite: 2D visualization

The 2D visualization page per gene shows the distribution of pathogenic and population missense variants’ positions across the protein sequence along with the protein feature information. The page is organized into seven subheadings for seven main protein feature categories, displaying the 2D plots of feature types (y-axis) of the amino acid residues (x-axis). The locations of variants in the plot are highlighted in colors by variant type. All the plots are readily downloadable. Hovering over the plots in the browser will display the residue position and corresponding amino acid. For large proteins or densely annotated regions, the user can zoom in or expand a specific segment of the protein using the ‘box’/‘lasso’ select capability to improve visualization. At any time, the axes can be reset using the ‘home’ icon in the control bar. These plots enable the user to analyze the missense variants in the context of features of residues or regions of functional interest.

For example, the distribution of *DDX3X* variants mapped on to the sequence along with the residues’ solvent accessible surface area (ASA) clearly illustrates that the residues with low ASA (<25 Å^2^) are frequently affected by pathogenic variants, perturbing the compact protein core which often leads to the destabilization of the structure (Figure 2C). On the contrary, the population variants tend to affect residues that are more exposed (>50 Å^2^ ASA).

### Variant analysis suite: 3D visualization

**Figure 3.**
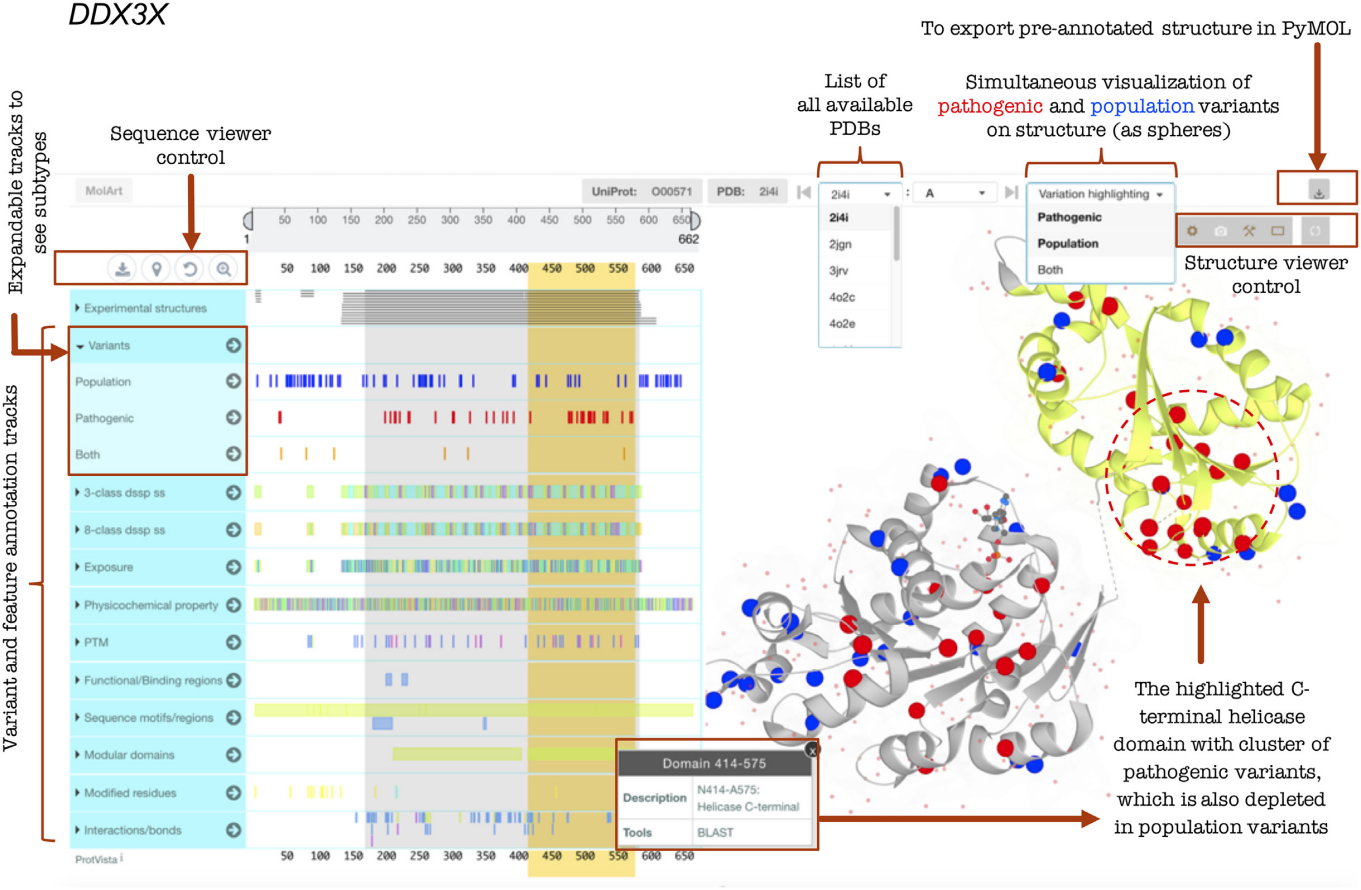
Illustration of MISCAST’s display of missense variants and protein feature annotations simultaneously on protein sequence (left panel) and structure (right panel). Mapping for pathogenic and population variants on the structure of *DDX3X* encoded ATP-dependent RNA helicase, along with highlighted (yellow) protein features annotation, shows a potential 3D mutational hotspot (cluster of pathogenic variants) in the C-terminal helicase domain.

Concurrent display of missense variants on protein sequence and structure in a common environment is one of the core capabilities of MISCAST, which is available per gene in the 3D visualization page. As an added dimension, all aggregated biologically relevant protein features are simultaneously overlaid on the structure (Figure 3). This allows users to visually inspect the features of pathogenic and population variant positions in the context of 3D structure.

For example, users can hover over a variant position on the sequence to highlight the corresponding position in the structure and vice versa, which can answer key questions such as: Is the variant perturbing a metal-interacting site or a ligand-binding pocket? The selection of a feature or variant from the sequence viewer shows a tooltip with details and highlights the corresponding annotation positions on structure. Besides residue wise investigation, users can select a full region or functional domain from the sequence viewer (e.g. the C-terminal helicase domain of DDX3X, Figure 3) and can visually inspect for the presence of clusters of pathogenic variants. The core capability of MolArt structure viewer is extended for MISCAST to highlight the C_α_ atoms of the amino acids as spheres. By selecting the pathogenic and population variants at the same time from the ‘Variation highlighting’ menu (Figure 3), the user can compare the spatial distribution of different variant types in structure. In the case of multiple available protein structures, users can select a specific structure of interest from the list of PDB identifiers (sorted alphanumerically, Figure 3). The structure viewer is expandable to perform in-depth analysis on the browser. And, importantly, the 3D structures with residue- and atom-wise (C_α_) annotations are exportable in PyMOL for advanced analysis, users, for example, can introduce new and in-house variants in the exported structure to investigate it in the context of known pathogenic and population variants and protein features.

### Variant analysis suite: feature table

Annotations of variants and features per amino acid are displayed in this page in a dynamic tabular format, where users can select/deselect a column of interest from the left-side menu to add to the table. Users can also view a selection of rows, e.g. only the residues harboring pathogenic or population variants. The ‘Download’ button on the top-left corner of the table will create of a local copy of the table as a tab-delimited text file.

### Variant analysis suite: index table

This page displays the mapping between the genomic index (format: ‘transcript>chromosome:strand:codon positions’) and structure index (format: ‘PDB id:chain:residue position’) of the amino acid residues of proteins. On the browser end, users can control the number of entries (or rows) to display in the table. Further, users can view the index mapping for the full protein or for parts thereof covered in the structure. The full table is also available to download, and will assist users to start analyzing a missense variant with known genomic position of the variant only, when necessary.

### Variant summary report

From the variant summary report track of MISCAST, users can obtain a text report for each amino acid residue of proteins summarizing the impact of the substitution of that residue. The report shows a pathogenic 3D feature index per residue based on the difference in it’s pathogenic and population variant-associated protein features (identified in an accompanying and separate study (32)). It is expected that the residues located in vulnerable 3D sites will have a higher number of pathogenic variant-associated features. These reports and indices are generated based on the features found to be enriched in pathogenic and population missense variants *in general* (‘All protein classes’) and separately for each annotated protein class for the query gene.

To illustrate an example, the submission of a gene name (e.g. *DDX3X*) on the top of the page will show a drop-down list of amino acid positions/indices. Selection or typing of a residue position (e.g. 235) will first list the known variants affecting that residue, e.g. the substitution of Leucine at position 235 (L235) to Proline is associated with intellectual disability. Then the summary report for L235 of *DDX3X* encoded nucleic acid binding protein will highlight that it possesses 6 pathogenic and 1 population variant-associated features as identified for the nucleic acid binding protein class in (32). This leads to a pathogenic 3D feature index value of 6 − 1 = 5 for L235. Users can obtain such summaries for their own set of variants, which importantly, can also be analyzed with respect to the variants’ protein features in structure from the Variant Analysis Suite track.

## DISCUSSION AND FUTURE DIRECTION

The number of identified missense variants and challenges in their interpretation are growing constantly. Currently, over 75% of all clinically-derived missense variants in ClinVar are of uncertain significance (33). To help eliminate this bottleneck in translational and clinical genetics and progress towards precision medicine, genomics or proteomics alone are not sufficient, but a bridging platform that can integrate data from genetics, molecular and structural biology is needed. MISCAST has been developed to address precisely this need.

The current version of MISCAST includes variants, pre-mapped on protein sequences and structures, from some of the largest catalogs such as gnomAD (5), ClinVar (6), HGMD (7) though is limited to 1330 disease-associated genes/proteins (about 6.5% of all human proteins, see Materials and Methods for selection criteria), and only considers sequence and structure of canonical protein isoforms. In contrast, tools such as PhyreRisk (17), VarMap (16) extend the mapping of variants to alternate isoforms and homologous structures. Other resources exist that provide a wide range of informative features which are complementary to MISCAST: PhyreRisk in association with missense3D (34) gives the predicted change in structure and free energy upon mutations, VarSite (15) presents the associated diseases, pathways, tissue specificity and affected organs, mutfunc (35) yields the mutation effect on structure stability and transcription factor binding sites.

Even if limited to a subset of all possible genes in the current version, the presented web server MISCAST provides the community with a valuable resource, that is, the parallel mapping and visualization of pathogenic and population missense variants on protein sequence and structure in an interactive web environment. Further integration of protein feature annotations (e.g. secondary structure type, residue’s solvent accessible surface area, protein-protein interactions, post-translational modifications, and functional site/regions) facilitates the quick investigation of the effect of variants in the context of protein structure and function, which, otherwise, would require the use of multiple tools and bioinformatics skills. In addition to the web-based platform, a unique capability of MISCAST is that the protein structures fully annotated with variants and features are freely downloadable for advance analysis in PyMOL, for example, alignment of the annotated structure with the homologous structures and explore variants on the aligned structures, calculation of the distance between mutation location and natural ligands.

We believe that MISCAST will serve as a useful resource for a diverse group of life scientists. For example, geneticists will benefit from the wealth of molecular data by easily exploring the variants on the structure and get valuable information (e.g. Is a variant of interest breaking a protein-stabilizing disulfide bond?). At the same time, structural biologists will benefit from the genomics data that will allow them to know, for example, if a mutation is present in a large fraction of the population with a high allele frequency. Such inclusive analysis using cross-disciplinary data and informative visuals as available in MISCAST will assist in informed variant interpretation, candidate variant selection for functional assays, and target selection for drug development. In the coming years, we aim to launch subsequent versions of MISCAST including an advanced set of protein annotations and increased coverage of the proteome, that is, all human proteins with available experimental structures and high-fidelity homology models (irrespective of the presence of a disease-associated variant, which is a filter criterion for the current version).
